# Strain Engineering of Unconventional Crystal-Phase Noble Metal Nanocatalysts

**DOI:** 10.3390/molecules29071617

**Published:** 2024-04-03

**Authors:** Jie Wang, Jiang Ye, Sixuan Chen, Qinyong Zhang

**Affiliations:** Key Laboratory of Fluid and Power Machinery of Ministry of Education, School of Materials Science and Engineering, Xihua University, Chengdu 610039, China

**Keywords:** phase engineering of nanomaterials (PEN), crystal phase, strain, nanocatalysts

## Abstract

The crystal phase, alongside the composition, morphology, architecture, facet, size, and dimensionality, has been recognized as a critical factor influencing the properties of noble metal nanomaterials in various applications. In particular, unconventional crystal phases can potentially enable fascinating properties in noble metal nanomaterials. Recent years have witnessed notable advances in the phase engineering of nanomaterials (PEN). Within the accessible strategies for phase engineering, the effect of strain cannot be ignored because strain can act not only as the driving force of phase transition but also as the origin of the diverse physicochemical properties of the unconventional crystal phase. In this review, we highlight the development of unconventional crystal-phase noble metal nanomaterials within strain engineering. We begin with a short introduction of the unconventional crystal phase and strain effect in noble metal nanomaterials. Next, the correlations of the structure and performance of strain-engineered unconventional crystal-phase noble metal nanomaterials in electrocatalysis are highlighted, as well as the phase transitions of noble metal nanomaterials induced by the strain effect. Lastly, the challenges and opportunities within this rapidly developing field (i.e., the strain engineering of unconventional crystal-phase noble metal nanocatalysts) are discussed.

## 1. Introduction

The crystal phase is a parameter referring to the periodic atomic arrangements of materials, which possesses great significance in determining their physicochemical properties and functionalities [1,2,3,4,5]. For example, diamond and graphite are both composed of carbon, but diamond, with its cubic lattice, is hard, while graphite, with its hexagonal lattice, is soft [6,7]. Furthermore, MoS_2_, with its hexagonal phase, is semiconducting, whereas MoS_2_, with its distorted octahedral phase, is semi-metallic [8]. Considering the chemical compositions of the aforementioned materials, the differences in their physicochemical properties should be ascribed to their different crystal phases.

Different from the conventional phase, defined as the thermodynamically stable phase of bulk materials under ambient circumstances, the unconventional counterpart, with a different atomic arrangement, is less thermodynamically stable because it possesses higher levels of free energy [9]; thus, it is difficult to attain the unconventional phase in the bulk state. However, if the size is decreased to nanoscale, the surface energy begins to play the dominant role and it is easier to prepare nanomaterials possessing unconventional phases using facile strategies [10,11,12,13]. As the unconventional phase could provide nanomaterials with novel catalytic, electrical, magnetic, and optical properties [14], the phase engineering of nanomaterials (PEN) is considered as a greatly promising route to maximizing the properties and functionalities of nanomaterials [7].

In various catalytic reactions, noble metal nanomaterials have attracted worldwide interests thanks to their high activity, robust chemical and structural stability, and favorable selectivity [15,16,17,18,19,20,21,22,23,24]. Benefiting from notable progress in the modulation of the size, facet, shape, composition, dimensionality, and architecture of noble metal nanomaterials, their properties can be rationally tuned for specific catalytic applications [25]. In addition, the crystal phase has been identified as another critical factor in modulating the electrical and catalytic properties of noble metal nanostructures [26]. For example, heterophase fcc-2H-fcc Au nanorods feature a faster electron exchange rate in electrocatalytic CO_2_RR than fcc Au nanorods or Au nanoparticles (NPs) [27]. Moreover, fcc-2H-fcc Pd@Au nanorods exhibit greater CO FEs than their fcc counterparts in CO_2_RR during CO production [28]. Furthermore, 4H PtCu nanostructures (4H-Au@4H-PtCu nanoribbons (NRBs)) exhibit better EOR mass/specific activity than their fcc counterparts (4H-Au@fcc-PtCu NRBs) [29]. Considering the significant effects of the crystal phase on the catalytic properties of noble metal nanomaterials, the phase engineering of noble metal nanomaterials is quite important. Regarding the optimization of the electrocatalytic properties of noble metal nanomaterials through phase engineering, the strain effect cannot be neglected as it can change the interatomic distances and influence orbital overlaps, thus resulting in variations in electronic structure. Specifically, the d-band center of transition metal atoms would be downshifted by compressive strain but upshifted by tensile strain [30,31], which would accordingly change the adsorption energy of the reaction intermediates on the transition metal atom’s surface [32]. Based on the Sabatier principle, catalytic activity is highly related to the adsorption strength of the reaction intermediates. If the adsorption strength is too low, the reaction cannot happen, while, if the adsorption strength is too high, the active sites would be covered by the intermediates and, thus, the catalytic activity would decrease. Moderate adsorption strength is best for catalytic activity, which can be accomplished by tuning the strain [33,34]. Hence, strain is important for surface reactivity. The effects of strain on the catalytic properties will also be discussed in the following sections. 

Furthermore, in spite of the effectiveness of optimizing the catalytic properties of noble metal nanomaterials via phase engineering, the phase engineering of noble metal nanomaterials is not easy, as harsh conditions (e.g., high temperatures and high pressures) are always required. Thanks to the rapid development of PEN, several strategies for the phase engineering of noble metal nanomaterials that do not require extreme conditions have been developed, including the direct synthesis method and phase transition between conventional and unconventional phases. Within the feasible methods for the phase engineering of noble metal nanomaterials, the strain effect should be highlighted because strain, caused by structural deformation, can alter the packing patterns and interatomic distances, as well as displacements or potential defects in crystals. As the packing mode has a significant effect on crystal structures, strain can be utilized as an effective tool for phase transformation [35]. The mechanisms for strain-modulated phase transformation will be specified in the following sections. Due to the critical effects of strain on both the catalytic properties and the phase transformation of unconventional-phase metallic nanomaterials, a number of key review articles have been published in recent years, which are listed in Table 1. 

In this review, the development, in strain engineering, of noble metal nanomaterials possessing unconventional phases towards phase transformation and the optimization of electrocatalytic activity will be briefly discussed. A brief introduction of the crystal phase of noble metal nanomaterials and the fundamentals of strain will be provided first in Section 2. Following this, the electrocatalytic performance of strain-engineered unconventional-crystal-phase noble metal nanomaterials in various reactions will be summarized in Section 3, focusing on their structure–performance relationships. Furthermore, the mechanisms of several types of strain-modulated phase transformation will be highlighted in Section 4. Lastly, the opportunities and challenges for the development of noble metal nanomaterials possessing unconventional phases by strain engineering will be discussed in Section 5.

## 2. Fundamentals of Unconventional Crystal Phase and Strain Effect

Recently, strain-engineered unconventional-crystal-phase nanomaterials have aroused widespread interest among researchers. To better understand the unconventional crystal phase and strain effect, it is indispensable to understand the unconventional crystal phase, strain and its origin, and how to evaluate strain. Thus, we introduce the definition of the unconventional crystal phase and the definition and characterization of strain in this section.

### 2.1. What Is the Unconventional Crystal Phase?

**Figure 1:**
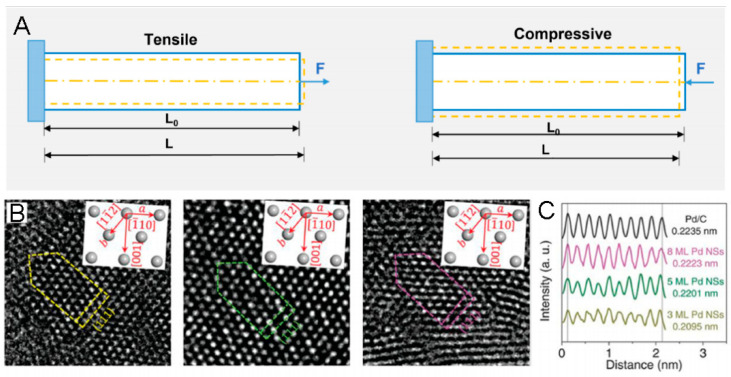
(**A**). Scheme of tensile and compressive strain. (**B**). AC-HRTEM images of Pd nanosheets (NSs) with different thicknesses on a carbon support. Insets in (**B**) show the three crystal directions of [1$\bar{1}$2], [$\bar{1}$10], and [001]. (**C**). Intensity profile and the corresponding d-spacings of Pd(111) planes of Pd NSs with different thicknesses in (**B**) and Pd/C [36].

Noble metals consist of Au, Pd, Ag, Pt, Os, Ru, Rh, and Ir; among them, Pt, Pd, Rh, Ir, Ag, and Au possess a face-centered cubic (fcc) phase and Ru and Os possesses a hexagonal closely packed (hcp, also known as 2H phase) phase, both of which are the thermodynamically stable/conventional phases of the corresponding noble metals. The atomic arrangements in the closely packed planes of the fcc and hcp phases are the same, while the stacking sequences of the closely packed planes of the fcc (“ABCABC”) and hcp (“ABAB”) phases are different [40]. Recently, the thermodynamically unstable/unconventional phases of noble metal nanomaterials have been achieved, such as the hcp-4H phase, where the stacking sequence of the closely packed planes follows the “ABCBABCB” mode [41,42,43]; the amorphous phase, which features long-range disordered arrangements with no closely packed planes (Figure 1) [44,45,46,47]; the face-centered trigonal phase and body-centered tetragonal phases in Au and Ag nanocrystals [48,49,50]; the fcc and 4H phases in Ru and Os nanocrystals [41,51,52,53,54,55]; and the 2H phase in Pd, Rh, Au, and Ag nanocrystals [1,56,57] (as listed in Table 2). 

### 2.2. What Is Strain?

Strain is a quantitative indicator of the deformation of a geometrical structure towards the tensile or compressive force applied, which is commonly calculated by the change in lattice spacings: strain = (L − L_0_)/L_0_ × 100%, where L represents the strained lattice spacing and L_0_ is the unstrained counterpart (Figure 1A) [35]. Progress in modulating the morphology, composition, and architecture in various nanomaterials enables researchers to engineer strain easily. Specifically, one of the most effective ways to modulate strain is to construct a core–shell architecture by epitaxial growth [12], surface dealloying [33], and galvanic replacement [63]. Benefiting from the lattice mismatch between the core and shell, lattice strain should be incurred in both the core and the shell, which decreases from the interface to the surface and in turn changes the electronic structure of the electrocatalyst. Another effective way to modulate strain is to tune the composition in the solid solution alloy [64]. By incorporating solute atoms possessing different lattice parameters in the solvent metal lattice, the lattice strain can be created. 

It is not easy to accurately evaluate strain and thus precise structural characterization is necessary. For example, XRD, as a common characterization tool for lattice spacing, can reveal the change in interplanar spacing by the positive or negative shift in the diffraction peaks [65]; thus, the lattice strain of nanocrystals can be evaluated by the experimental value and theoretical or reference counterparts. In contrast to XRD, extended X-ray absorption fine structure (EXAFS) analysis, developed with synchrotron radiation, is sensitive to the localized structure near the absorbing atoms, without a dependence on the long-range-ordered structure [66], which can be utilized to resolve specific elements [67], analyze amorphous materials [68], and demonstrate the strain effect by the variation in the bond length in the R space. For example, the controllable compression of IrOx with diverse thicknesses on an IrCo core was confirmed by the change in the Ir-O bond length based on the EXAFS of the Ir L3 edge [69]. 

By comparison with the above techniques, HRTEM is the most direct tool to obtain the lattice parameters of some local areas. Specifically, the change in the lattice parameters of Pd nanosheets (NSs) with diverse layers, i.e., the correspondingly modulated compressive strain, can be observed by HRTEM (Figure 1B,C) [70]. It is worth noting that atomic resolution HRTEM can be used to measure the strain field; for example, geometric phase analysis (GPA), developed by the Hytch group, is capable of mapping the strain distribution of a localized area [71]. Moreover, by taking advantage of the strain mapping, the strain state of a decahedral Au nanoparticle can be quantitatively studied [72]. However, considering the 3D quality of lattice strain, 2D GPA is not able to reflect all of the structural information. Thus, a 3D measurement method, on the basis of high-resolution electron tomography reconstructions, is proposed, which can be used to obtain a more significant 3D displacement map of the Au nano-decahedron [73]. Furthermore, 4D STEM has been developed as a more advanced technique for strain calculation [74], by which more precise strain distributions and evolution in nanomaterials can be facilely observed. 

Benefiting from the above-mentioned advanced techniques, the strain in nanomaterials can be comprehensively characterized, and this is important in investigating the strain effect of nanomaterials, which is highly related to the catalytic activity of nanomaterials.

## 3. Structure–Performance Relationship of Strain-Engineered Unconventional-Crystal-Phase Noble Metal Nanomaterials

The strain effect on catalytic activity should be ascribed to the synergy of the geometry effect and electronic effect, i.e., the strain of lattices in nanocrystals can change the interatomic distance, leading to a variation in the orbital overlaps and thus the electronic structure correspondingly. Specifically, compressive strain can broaden the d-bands of transition metals and the d-band center should move down relative to the Fermi level to conserve the d-band occupancy; in contrast, tensile strain narrows down the d-bands of transition metals and thus causes the d-band center to move up [30,31]. The variation in the d-band center is highly related to the adsorption strength of the intermediate species of catalytic reactions on the nanocatalyst’s surface [32]. According to the Sabatier principle, a weak adsorption strength is not favorable for the reactant’s activation, while a high adsorption strength is not beneficial to product desorption, causing the active sites of the catalysts to be covered. Both situations are not favorable to the electrocatalytic activity and hence there should be a volcano relationship between the catalytic activity and the adsorption strength. Overall, strain tuning is important for the optimization of catalytic activity and has been thoroughly studied in conventional-crystal-phase catalysts, which are always metastable and are more active in various catalytic reactions [33,34]. However, little attention has been paid to the strain effect and strain tuning of unconventional-crystal-phase catalysts. In this section, the structure–performance relationships of unconventional-crystal-phase noble metal nanomaterials are specified and the strain tuning methods are also highlighted.

### 3.1. Oxygen Reduction Reaction (ORR) 

A fuel cell is a clean energy device for sustainable development, whose performance is limited by the sluggish reaction rate of its cathode reaction—the oxygen reduction reaction (ORR). The ORR is a favorable model to study the strain effect of nanocatalysts on the mass and specific activities by tracking the variation of the bonding energy of the reaction intermediates, including *H, *OH, and *OOH [61,75,76,77,78].

**Figure 2:**
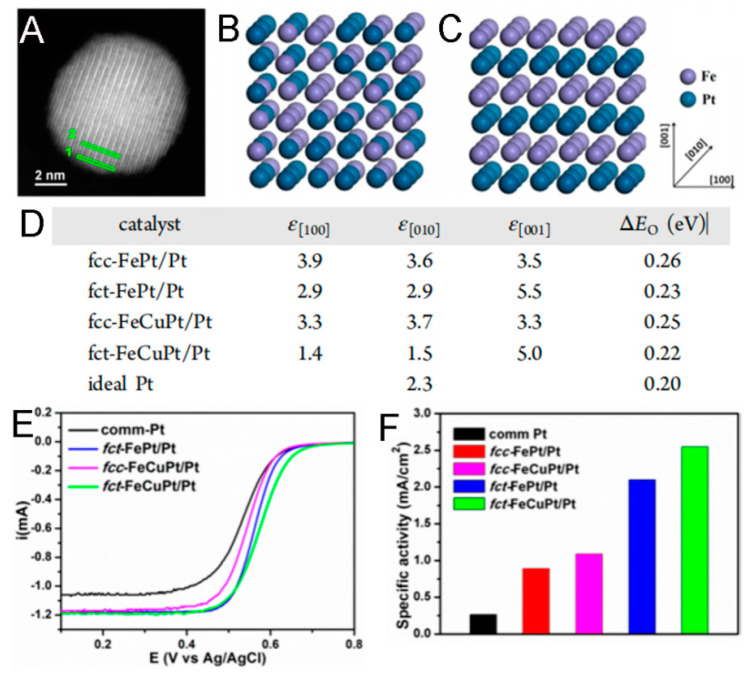
(**A**). HAADF−STEM image of a typical fct-FeCuPt/Pt nanoparticle. The periodic super cells of fcc−Fe50Pt50 (**B**) and fct−Fe50Pt50 (**C**) used to calculate the crystalline lattice constants. (**D**). Surface strain (ε, in %) from DFT calculations and ΔEO from QM-MM simulations. ORR polarization curves (**E**) and specific activities of Pt, fct-FePt/Pt, fcc-FeCuPt/Pt, and fct-FeCuPt/Pt catalysts (**F**) [61].

For example, Zhang et al. prepared fct-FePt/Pt core–shell NPs via the epitaxial growth method and found that the fct-Pt exhibited better ORR activity than fcc-Pt because of the Pt surface strain modulated by the fct-PtFe core (Figure 2A–D) [61]. Moreover, the Pt strain effect could be modulated by the partial Cu replacement of Fe, leading to the maximized ORR activity of FeCuPt/Pt NPs (Figure 2E,F). Furthermore, as suggested by a DFT calculation work from Prof. Wu’s group, the superior ORR activity of Pt-based L10 fct core–shell NPs should be ascribed to the distinct shear strain in the tetragonal core [79]. Additionally, the fct phase can also bring about better durability in the catalysts mentioned above.

Similar to the Pt-based nanocatalysts with the fct phase, Pd-based nanocatalysts with the fct phase can also show superior ORR performance thanks to the strain effect. For instance, Maiti et al. synthesized fct-PdFe@Pd NPs deposited on N-doped graphene and noticed a synergistic effect aroused by the PdFe core and Pd shell, inducing a compressive lattice strain and modulating the electronic structure of the Pd surface, which enhanced the ORR activity by decreasing the adsorption strength of the oxygenated intermediates [80]. 

Beyond the fct phase, Pd-based nanocatalysts with other unconventional phases have also demonstrated distinct strain effects from their conventional counterparts. For example, the conventional crystal phase of Pd5Ce is a low-temperature phase (L-Pd5Ce), featuring a cubic symmetry, and the unconventional crystal phase of Pd5Ce is a high-temperature phase (H-Pd5Ce), featuring a hexagonal symmetry. When a Pd shell is deposited on L-Pd5Ce, the Pd shell should be exposed to tensile strain, and when a Pd shell is deposited on H-Pd5Ce, the Pd shell should be exposed to compressive strain. In ORR tests, the L-Pd5Ce with expanded Pd lattices on the surface showed inferior ORR activity to the polycrystalline Pd, matching well with the DFT results [81]. 

Overall, the ORR activity can be optimized by the modulated adsorption strength of oxygen-containing intermediates, which is influenced by both the unconventional phase and the strain effect.

### 3.2. Hydrogen Evolution Reaction (HER) 

Similar to ORR, HER performance is also highly related to the adsorption strength of the reaction intermediates, such as *H and*OH, which is greatly influenced by the strain effect too. Thus, an appropriate adsorption strength is indispensable to maximize the HER performance by controlling the strain [82,83]. 

**Figure 3:**
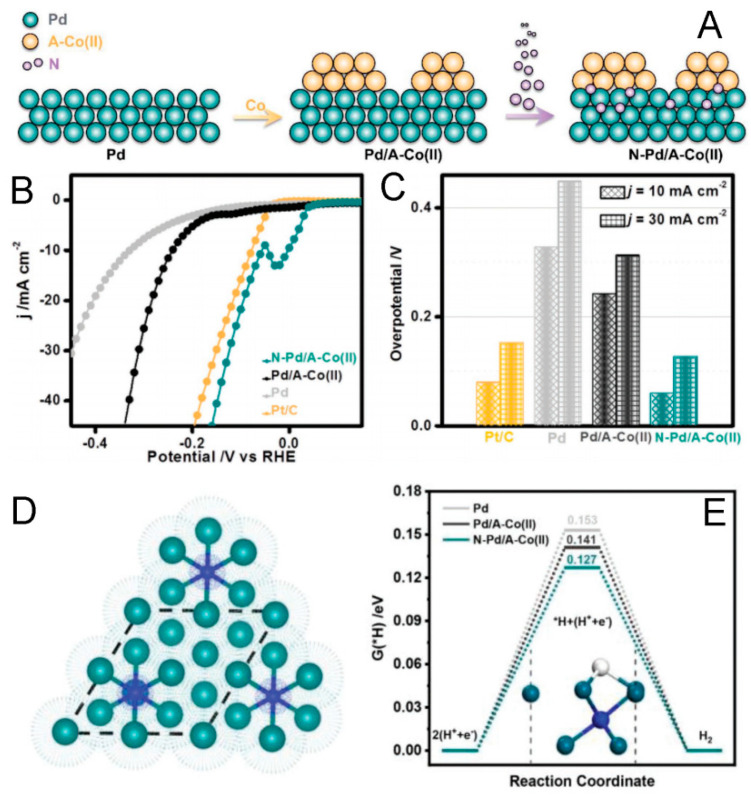
(**A**). Scheme of the N-Pd/A-Co(II) structure with constructed strain interface. LSV curves (**B**) and overpotentials at different current densities (**C**) of N-Pd/A-Co(II), Pd/A-Co(II), Pd, and Pt/C in 1.0 m KOH solution. (**D**). Top view of N-doped Pd(111) surface (green and blue balls correspond to Pd and N atoms, respectively). (**E**). Strain effect of N-doped Pd(111) on the hydrogen adsorption free energy [84].

For example, Shao’s group constructed a N-Pd/A-Co(II) nanostructure with a compressive interface between the N-doped Pd core (N-Pd) and amorphous Co(II) shell (A-Co(II)) (Figure 3A), which exhibited a much lower overpotential (58 mV) than both the pure Pt/C (78 mV) and Pd (327 mV) at 10 mA cm^−2^ (Figure 3B,C) and a negligible activity decay after a 30-h stability test in 1.0 M KOH [84]. The compressive strain, derived from the core–shell interface, could be clearly revealed by the apparently shifted (111) peak of N-Pd/A Co(II) in contrast to those of Pd/A-Co(II) and pure Pd, which could downshift the d-band center of Pd and weaken the H adsorption strength on the Pd surface (Figure 3D,E). Additionally, the amorphous Co(II) shell can facilitate water dissociation. Thanks to the synergistic effect of the compressed N-doped Pd surface and the amorphous Co(II) shell, a remarkable HER electrocatalyst was thereby developed.

This impressive strain effect was also observed in the 2H-fcc-2H Pd_45_@Ir_55_ multibranched nanodendrite core–shell structures constructed by Ge et al. by phase-selective epitaxial growth on 2H-Pd seeds, which exhibited only 11.0 mV overpotential, much lower than those of the commercial Pt/C and Ir/C, at 10 mA cm^−2^ in an acidic environment [62]. This superior HER performance was due to the optimized H adsorption on the catalyst surface, derived from the corresponding electronic structure modified by the unconventional fcc-2H-fcc heterophase Ir and the internal strain. The Ir 4f XPS spectra indicated that the Ir 4f peaks of the fcc-2H-fcc heterophase Pd_45_@Ir_55_ evidently shifted to higher binding energies by comparison with those of the fcc counterparts (Pd_47_@Ir_53_), indicating the different electronic structures of Ir in the fcc-2H-fcc heterophase Pd_45_@Ir_55_ catalyst. Furthermore, the electronic structure could be also tuned by the internal strain resulting from the lattice mismatch between the 2H-Pd core and 2H-fcc-heterophase Ir shell, as evidenced by the apparent shift in the diffraction peaks in the XRD pattern.

Briefly, the strain effect of unconventional-crystal-phase nanocatalysts can modify the electronic structures of the catalysts and thereby optimize the adsorption energy of the intermediates such as *H on the surface, leading to improved HER performance.

### 3.3. CO_2_ Electroreduction (CO_2_RR)

The HER is not always favorable, especially as it can act as a competitive reaction for the electroreduction of CO_2_ (CO_2_RR), which can transfer CO_2_ into valuable chemicals (such as CH_4_, C_2_H_4_, HCOOH, etc.) by electrical energy under mild conditions (normal temperature and pressure). As CO_2_RR can not only complete the closure of the artificial carbon cycle but also realize the synthesis of high-value chemicals [85], it is meaningful from not only a scientific but also an economic view. Thus, the development of robust electrocatalysts for CO_2_RR is a current hot topic [86].

**Figure 4:**
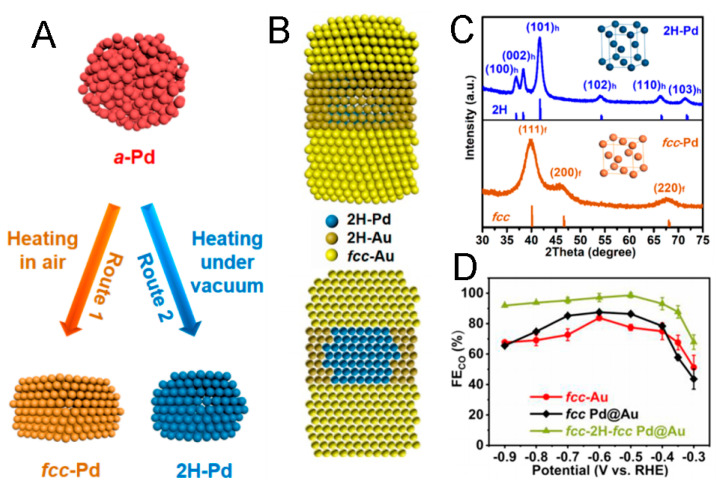
Scheme of the phase transformation from amorphous Pd to fcc-Pd (route 1) and 2H-Pd (route 2) NPs (**A**), the structure of heterophase Pd@Au nanorods (**B**). (**C**) XRD spectrum and models of fcc-Pd and 2H-Pd NPs. (**D**) CO FEsof fcc-2H-fcc Pd@Au nanorods, fcc Pd@Au NPs, and fcc-Au nanorods [28].

For example, by the controlled phase transition of amorphous Pd NPs, 2H-Pd NPs can be prepared (Figure 4A,C), and by using the 2H-Pd NPs as seeds, fcc-2H-fcc heterophase Pd@Au core–shell nanorods, with fcc-Au grown on the (002)_h_ facets of 2H-Pd and 2H-Au grown on the other exposed facets of 2H-Pd, can be obtained (Figure 4B), which show outstanding CO_2_RR activity for CO production, with >90% Faradaic efficiency from −0.9 to −0.4 V (vs. RHE) (Figure 4D) [28]. The excellent activity can be ascribed to two aspects: (1) the lattice mismatch between the Pd core and Au shell can result in internal strain [87], as confirmed by the shifted XRD peaks of Pd@Au in contrast to fcc-Au, which can evidently improve the CO_2_RR performance towards CO production [88]; (2) the existence of 2H/fcc phase boundaries can also lead to enhanced CO_2_RR performance because 2H/fcc hetero-phase Au nanostructures feature a lower kinetic barrier for the electrochemical CO_2_RR towards CO production than that of the fcc Au [27]. Hence, both the strain and heterophase structures of fcc-2H-fcc Pd@Au nanorods are critical to their outstanding selective CO_2_RR performance to CO.

Other than the performance improvement, strain can also stabilize the metastable unconventional phase [89,90,91,92]. For example, Yu et al. obtained fct Au deposited on ordered intermetallic AuCu_3_ NPs (o-AuCu_3_@fct Au) and the unconventional fct Au nanostructure was stabilized by the interface strain, as confirmed by the edge dislocations between the core and shell, derived from the lattice mismatch between the fct Au and ordered intermetallic AuCu_3_, which can be observed in the AC HAADF-STEM image [58]. The o-AuCu_3_@fct Au featured higher Faraday efficiency (FE) as well as specific activity towards CO production than the fcc Au counterparts, without a noticeable activity decay during a 20-h stability test, which could be ascribed to the lowered energy barrier for the production of intermediate (COOH*) by the upshifted d-band center arising from the metastable fct Au. Although the interface strain cannot facilitate electro-transfer during the reaction process, it can contribute to the stabilization of this unconventional metastable fct phase, which leads to the remarkable performance. 

In short, strain can not only participate in the activity enhancement due to its effect on the electronic structure, but also can stabilize the metastable phase, both of which are favorable towards the impressive performance of CO_2_RR.

### 3.4. Alcohol/Formic Acid Oxidation Reaction (AOR/FAOR)

Electrochemical oxidation reactions of small molecules such as methanol, ethanol, and formic acid always refer to two different pathways. The first one is a direct pathway, meaning that the small molecule is converted to CO_2_ without the generation of poisonous intermediates, and the second one is an indirect pathway, meaning that CO, instead of CO_2_, is produced, which may deactivate the catalyst [93]. To achieve favorable AOR and/or FAOR performance, researchers have devoted great efforts to developing catalysts with abundant active sites, and the appropriate adsorption of intermediates is indispensable.

**Figure 5:**
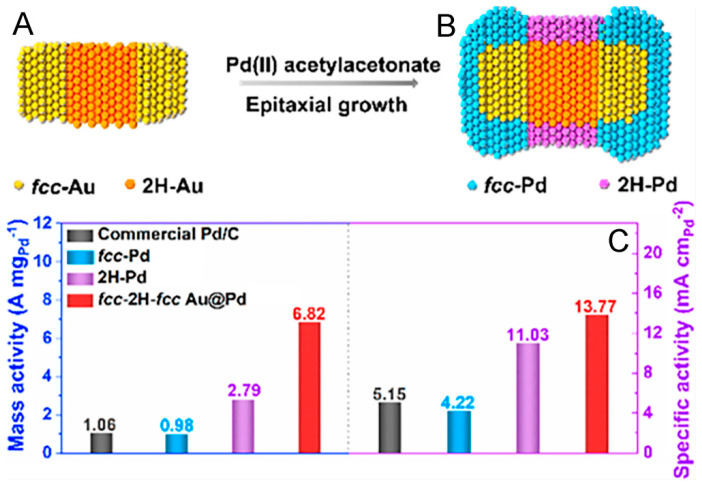
Scheme of the structure of fcc-2H-fcc Au heterophase nanorod (**A**), and the preparation of fcc-2H-fcc heterophase Au@Pd nanorod by epitaxial deposition of Pd ions on fcc-2H-fcc Au surface (**B**). (**C**) Mass and specific activities of commercial Pd/C, fcc-Pd NPs, 2H-Pd NPs, and fcc-2H-fcc heterophase Au@Pd nanorods [96].

For example, Zhou et al. constructed fcc-2H-fcc heterophase Au@Pd core–shell nanorods (Figure 5B) by epitaxially depositing Pd atoms on the fcc-2H-fcc Au (Figure 5A) surface, leading to tensile strain in the Pd shell owing to the lattice mismatch between the Au core and Pd shell [94]. The fcc-2H-fcc heterophase Au@Pd nanorods, preferring the C2 pathway, featured not only favorable activity (Figure 5C) but also even improved the stability towards EOR, in contrast to the 2H Pd, which could be attributed to the decreased energy barrier for *CH_3_COOH formation by the expanded lattices in the 2H(110)/fcc(101) phase boundary, according to the DFT simulation result.

As another example, amorphous Pd, constructed by the phosphorization of fcc Pd nanocubes, features flexible structures, which can be used to reversibly regulate the strain of Pt lattices epitaxially grown on the Pd nanocubes [95]. Specifically, Pt atoms were epitaxially deposited on the fcc Pd nanocube surface by He et al., and tensile strain could be introduced into the Pt shell by P insertion to the Pd core, which was converted to amorphous Pd with an expanded volume. Similarly, compressive strain can be introduced into the Pt shell by the deposition of Pt atoms on PdP nanocubes first, followed by the dephosphorization of the PdP core with a reduced volume. Overall, by inserting and extracting P, the Pd core was expanded and diminished, modulating the strain in the Pt shell from −5.1 to 5.9%. Both the tensile and compressive strain in the Pt shell were proven to benefit MOR performance via the M-shaped curve of MOR activity, with the two peaks achieved at −3.9% and 4.7% strain, respectively. According to DFT simulations, tensile strain can upshift the d-band center of Pt, benefiting OH- production and thereby CO* release (CO* + OH* = CO_2_ + H^+^ + e^−^). In contrast, compressive strain can downshift the d-band center of Pt, decreasing the CO* binding. As a result, the MOR activity can be boosted due to the promoted CO removal by either tensile or compressive strain.

In brief, MOR electrocatalysts require a suitable adsorption strength of carbon-containing intermediates, which can be achieved by strain engineering towards nanocatalysts with unconventional crystal phases.

From the above work, we can determine that strain engineering is important in modulating the electronic structures of electrocatalysts and thereby tuning the adsorption strength of the intermediates of catalytic reactions, which should be neither too strong nor too weak. The optimized electrocatalytic performance of unconventional-crystal-phase noble metal nanocrystals when regulating the strain is summarized in Table 3. Moreover, we can note the importance of the crystal phase in regulating the catalytic activity in this section, considering that catalysts with metastable phases always feature a lower kinetic barrier and are thus more active towards various catalytic reactions. Therefore, the modulation of the crystal phase is important.

## 4. Strain-Assisted Phase Transformation

As mentioned above, phase modulation is crucial as the crystal phase is important to the physicochemical properties of nanocatalysts. Besides the effect on catalytic activity, as introduced in Section 3, strain is also a critical driving force to facilitate the phase transformation of noble metal-based nanocatalysts by bringing about variations in the interatomic interaction, packing patterns, and defects, which can be achieved through template-mediated growth, surface ligands, and external pressure. This is because the surface state becomes increasingly important to the materials’ stability when the size of the materials decreases and the surface atom proportion increases. Thus, strain modification is an effective way to control the structure transition of metastable nanomaterials with unconventional crystal phases. This section will introduce the mechanisms of the strain-assisted phase transition through a case study. 

### 4.1. The 4H-to-fcc Phase Transformation

In 2015, Fan et al. firstly prepared 4H Au NSs and found different SPR properties in 4H Au in contrast to fcc Au [3]. Since then, great effort has been devoted to investigating the physicochemical properties of 4H Au and more intriguing differences between 4H and fcc Au have been noticed [7,10,12]. Thus, the phase engineering between 4H and fcc is of significance not only to their study but also their application. 

**Figure 6:**
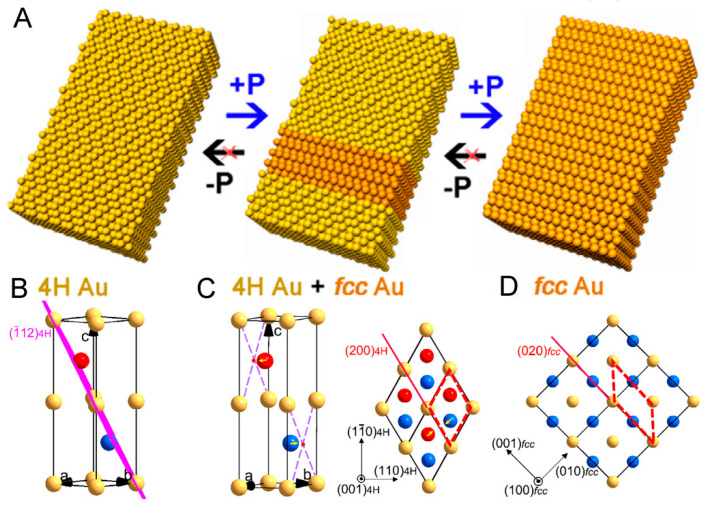
Mechanism of phase transformation caused by high pressure. Scheme of the phase transformation of Au nanoribbon from 4H to fcc (**A**), 4H unit cell (**B**), the 4H to fcc phase transition in 4H unit cell (**C**), and the 4H to fcc phase transition viewed along the (001)_4H_ (left) and (100)_fcc_ planes (right) (**D**) [98].

It is worth noting that Li et al. observed the 4H-to-fcc phase transition of Au NRBs by applying high pressure to the original 4H Au NRBs (Figure 6) [98]. Normally, in response to high pressure, the crystals should be deformed to other structures or even other shapes, and, through this process, the crystal lattices should be strained. The shrinkage of the 4H Au crystal lattice was revealed in the XRD patterns, and the decreased intensities of the 4H peaks along with the increased intensities of the fcc peaks indicated the gradual 4H-to-fcc phase transition process. By combining first-principle calculations with the experimental results, they proposed an atom-based transformation mechanism in which the atoms should shift from the (112) facets of 4H Au to the nearest face centers of the four nearest Au atoms in the ac plane towards the strain induced by high pressure (Figure 6B–D).

Furthermore, benefitting from the strain, the metals that are normally crystallized in the fcc phase can be crystallized in the 4H phase. For example, by using epitaxial growth methods with 4H-Au NRBs as templates, a wide range of monometallic (e.g., Ir, Pt, Ru, Pd, Ag, Rh) and multimetallic nanostructures (e.g., PdAg, PdFe, PdRu, PdIr, PtAg, PtCu, PtCo, RuRh, PtPdAg) with the 4H phase have been obtained [29,41,63,96]. In these cases, the lattice mismatch, arising from the different crystal structures of the core and shell, should deform the crystal structure of the shell and thereby provoke the occurrence of strain, which can play the role of a constraint towards the shell geometry, causing the shell to grow in the same crystal phase as the core. 

### 4.2. The 2H-to-fcc Phase Transformation

**Figure 7:**
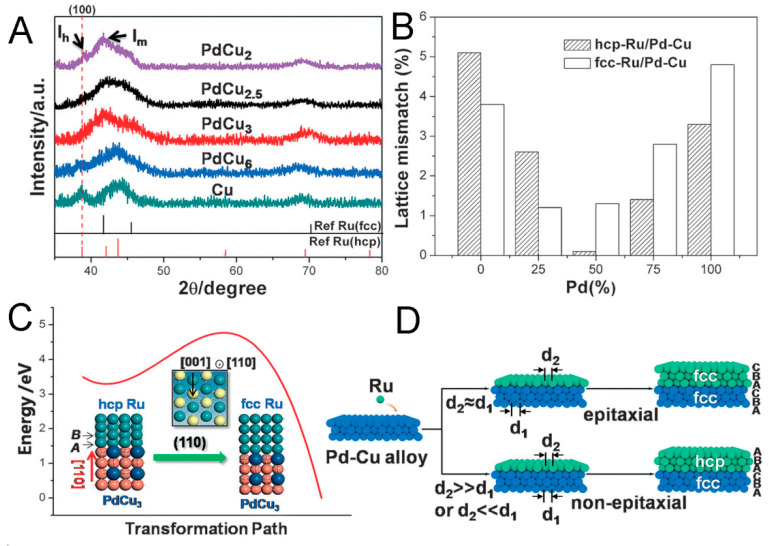
(**A**). XRD spectra of Pd-Cu@Ru core–shell NPs with different Pd/Cu ratios. (**B**). Lattice mismatch between Ru crystallized in hcp or fcc phase and PdXCu alloys. (**C**). The 2H-to-fcc phase transformation path of Ru on PdCu alloy, promoted by the elastic energy (strain effect) aroused from lattice mismatch. (**D**). Scheme of the Ru (hcp and fcc) grown on Pd-Cu alloy [52].

Interestingly, by controlling the lattice mismatch between the core and shell, the strain in the system can be modulated and thereby the 2H-to-fcc phase transformation can be tuned by changing the growth pattern (epitaxial or non-epitaxial growth). Specifically, Ru is commonly crystallized in the 2H crystal phase. By using PdCu nanocrystals as templates, fcc Ru can be obtained by epitaxially growing it on PdCu_3_ and PdCu_2.5_ because of their relatively small lattice mismatch compared to fcc Ru (Figure 7A). For other PdCu templates possessing a higher or lower Pd/Cu atomic ratio, with increased lattice mismatch to generate sufficient strain, the epitaxial relationship between PdCu and Ru should be eliminated (Figure 7B) and then hcp Ru would be dominant, because Ru prefers the global energy minimum geometry structure (Figure 7C,D) [52].

Besides the 2H-to-fcc phase transformation of Ru, the 2H-to-fcc phase transformation of Au could be controlled as well. For example, Zhang’s group succeeded in obtaining ultrathin 2H Au NSs by using GO as templates [1] and the 2H-to-fcc phase transition of the 2H Au could be achieved by the epitaxial growth of Pt or Pd [99], which can be interpreted by the following aspects: (1) the Pt (or Pd) shell made the Au@Pt (or Pd) NSs thicker, leading to a decrease in the ratio of surface to volume energy, which destabilized the 2H phase; (2) the small energy difference between the 2H and fcc phases [100]; (3) considering that 7.5 monolayers of ZnS can give rise to radial pressure of up to 4 GPa towards the CdS core in CdS@ZnS core–shell NPs (with 7% lattice mismatch between the core and shell) [101], the strain effect of the Pt shell on the Au core (with 5.8% lattice mismatch between the core and shell) should be significant. 

It is worth noting that, other than the metal-coating-induced phase transformation of 2H Au NSs, ligand exchange could be used to facilitate the 2H-to-fcc phase transformation as well. Fan et al. demonstrated that thiol molecules can adsorb on the 2H Au surface and cause 2H-to-fcc phase transition through surface reconstruction. In this case, the phase transformation should be ascribed to the strong interaction between the ligand and metal surface, which can result in strain on the adsorption sites [102] and thereby deform the surface structure. Thus, ligand adsorption is an effective method for phase transformation and strain is the dominant factor in the whole transformation process. 

Other types of phase transformation, such as the fcc-to-amorphous phase transition of Pd nanocrystals [2], fcc-to-bct phase transition of Au and Ag nanostructures [49,103], and fcc-to-fct phase transition in Pd nanocubes [60], have been achieved. Importantly, for all of these types of phase transformation, strain acts as the dominant driving force by influencing the surface energy of the nanostructures. Note that, differently from the bulk state, for nanoscale systems, the surface energy, instead of the volume energy, determines the phase stability. Thus, the key factor for phase modulation is strain control, through the template-mediated growth, ligand exchange, and external pressure methods mentioned above.

## 5. Summary and Outlook

The crystal phase is an important factor in controlling the physicochemical properties of nanomaterials. An unconventional crystal phase, possessing different atomic arrangements in contrast to its conventional counterparts, is thermodynamically metastable because of its higher free energy, and it is able to endow nanomaterials with unconventional catalytic, electrical, magnetic, and optical properties. For various catalytic reactions, noble metal nanomaterials are favorable catalysts due to their high activity, robust stability, and expected selectivity, all of which can be influenced by the crystal phase. Thus, the phase engineering of noble metal nanomaterials is quite important. 

In order to maximize the catalytic properties of noble metal nanomaterials by phase engineering, the effect of strain should be highlighted because strain can change the lattice spacing, influence the electronic structure, control the surface adsorption strength, and further tune the catalytic performance. Thus, the structure–performance relationships of strain-engineered unconventional-crystal-phase noble metal nanocatalysts deserve systemic study, which can improve the understanding and design of noble metal nanocatalysts towards target catalytic reactions. 

The phase engineering of noble metal nanomaterials is effective in optimizing their catalytic properties, but it is not easy and it always requires harsh conditions (e.g., high temperature and high pressure). Phase transformation is a practical means of phase engineering, without the need for harsh conditions. For the viable methods of phase transformation, the strain effect cannot be ignored because strain can cause structural deformation and change the surface energy, which always plays the role of the driving force for phase transformation.

In spite of the above-mentioned achievements in this fast-developing field, some major challenges still remain.

1. Currently, the strain effect of noble metal nanocatalysts with different crystal phases, consisting of conventional or unconventional crystal phases, has been studied. However, the strain effect aroused by the strain imposed on different facets of unconventional-crystal-phase noble metal nanocatalysts has not been widely investigated. To study this issue, the prerequisite is to synthesize various shapes of unconventional-crystal-phase noble metal nanocatalysts covered by different facets. Taking 4H Au as an example, until now, only 4H Au NRBs covered by {110}_4H_ facets have been prepared. The preparation of 4H Au nanostructures enclosed by {100}_4H_ or {111}_4H_ facets would be not only interesting but also significant in solving this issue.
2. At present, only a few types of unconventional crystal phases in specific noble metal nanostructures have been synthesized, such as fcc Ru, fct PtFe, amorphous and 2H Pd, 2H, and 4H Au. It is critical to synthesize 4H Ru, Pt, Pd, and amorphous Pt, Ir, and Os, among others, in facile one-pot methods, instead of epitaxial growth methods, to comprehensively and systemically study the structure–performance relationships of the unconventional-crystal-phase noble metal nanocatalysts.
3. Considering the fact that noble metal nanocatalysts with unconventional crystal phases feature metastable crystal structures and the original crystal phase may be changed by the catalysis reaction due to the adsorption of the reaction intermediates, in situ technology and characterization are indispensable to elaborate the structure–performance relationships of the unconventional-crystal-phase noble metal nanocatalysts towards the specific catalytic reaction.
4. An understanding of the phase transformation is still lacking. Theoretical simulations would be helpful to determine the phase transformation mechanism and thereby give some general principles to rationally construct the target crystal phase.

It is reasonable to anticipate that great progress could be achieved by solving these challenges. 

## Figures and Tables

**Table 1:** Key review articles about strain-engineered unconventional-phase metallic nanomaterials.

Topic	Journal	Year	Ref
Strain and Surface Engineering of Multicomponent Metallic Nanomaterials with Unconventional Phases.	*Chem. Rev.*	2023	[36]
Structural Transformation of Unconventional-Phase Materials.	*ACS Nano*	2023	[37]
Colloidal Metal Nanocrystals with Metastable Crystal Structures.	*Angew. Chem. Int. Ed.*	2021	[13]
Phase Engineering of Nanomaterials.	*Nat. Rev. Chem.*	2020	[5]
Strain Modulation of Phase Transformation of Noble Metal Nanomaterials.	*InfoMat*	2020	[35]
Strain Engineering of Metal-based Nanomaterials for Energy Electrocatalysis	*Chem. Soc. Rev.*	2019	[38]
Strain-Controlled Electrocatalysis on Multimetallic Nanomaterials.	*Nat. Rev. Mater.*	2017	[39]

**Table 2:** Conventional and unconventional crystal phases of noble metal nanomaterials.

Elements	Conventional Phases	Unconventional Phases
Au	fcc	2H [1], 4H [3], bct [50], bco [50], fct [58]
Ag	fcc	2H [57], 4H [29], bct [48], fct [49], trigonal [59]
Pd	fcc	2H [28], 4H [43], amorphous [2], fct [60]
Pt	fcc	fct [61]
Ru	2H	fcc [51,52,53], amorphous [45]
Rh	fcc	2H [56], amorphous [45]
Os	2H	4H [41]
Ir	fcc	4H [41], 2H [62], amorphous [44]

**Table 3:** The optimized electrocatalytic performance of unconventional-crystal-phase noble metal nanocrystals by regulating the strain.

Electrocatalytic Reaction	Catalyst	Performance		Ref.
		Mass Activity	Specific Activity	
ORR	fct-FeCuPt/Pt NPs	N/A	2.55	[61]
	2H-PdCuPt	7.00 A/mg @ 0.85 V	N/A	[97]
HER	fcc-2H-fcc Pd_45_@Ir_55_	1.16 A/mg	3.55 mA/cm^2^	[62]
	4H/fcc Au-Ru NWs	N/A	0.35 mA/cm^2^	[87]
CO_2_RR	fcc-2H-fcc Pd@Au NPs	93% (Faradaic efficiency) @−0.4 V 97% (Faradaic efficiency) @−0.6 V 92% (Faradaic efficiency) @−0.9 V		[28]
	Ordered intermetallic AuCu_3_@fct Au	94.5% (Faradaic efficiency) @−0.8 V		[58]
AOR	4H-Au@PtCu NRBs	4.22 A/mg	50.2 mA/cm^2^	[29]
	4H-Au@PdFe NRBs	3.69 A/mg	23.6 mA/cm^2^	[96]
	fcc-2H-fcc Au@Pd nanorods	1.14 A/mg	N/A	[94]

## Data Availability

Not applicable.
